# Congenital hypoplasia of the posterior arch of the atlas accompanied with cervical high-intensity intramedullary lesions: a case report

**DOI:** 10.1093/jscr/rjae689

**Published:** 2025-04-22

**Authors:** Jian Zhang, Jiahao Liu, Jingyu Jia, Xinxin Miao, Xigao Cheng

**Affiliations:** Department of Orthopedics, The Second Affiliated Hospital, Jiangxi Medical College, Nanchang University, No. 1 Minde Road, Nanchang, Jiangxi 330006, China; Institute of Orthopedics of Jiangxi Province, No. 461 Bayi Avenue, Nanchang, Jiangxi 330006, China; Department of Orthopedics, The Second Affiliated Hospital, Jiangxi Medical College, Nanchang University, No. 1 Minde Road, Nanchang, Jiangxi 330006, China; Department of Orthopedics, The Second Affiliated Hospital, Jiangxi Medical College, Nanchang University, No. 1 Minde Road, Nanchang, Jiangxi 330006, China; Department of Orthopedics, The Second Affiliated Hospital, Jiangxi Medical College, Nanchang University, No. 1 Minde Road, Nanchang, Jiangxi 330006, China; Department of Orthopedics, The Second Affiliated Hospital, Jiangxi Medical College, Nanchang University, No. 1 Minde Road, Nanchang, Jiangxi 330006, China; Institute of Orthopedics of Jiangxi Province, No. 461 Bayi Avenue, Nanchang, Jiangxi 330006, China; Jiangxi Provincial Key Laboratory of Spine and Spinal Cord Disease, No. 461 Bayi Avenue, Nanchang 330006, Jiangxi, China

**Keywords:** atlas, cervical high-intensity intramedullary, congenital, hypoplasia, posterior arch

## Abstract

Congenital hypoplasia of the posterior arch of the atlas (C1) is uncommon and typically asymptomatic. A 49-year-old woman presented with a 2-year history of recurrent vertigo attacks. One month before admission, she began to have weakness in both lower extremities and unsteady walking, with chest tightness and shortness of breath after walking. She did not have any history of trauma. Cervical spine imaging revealed partial hypoplasia of the posterior arch of C1, midline cleft of the anterior arch, and cervical high-intensity intramedullary lesions. We used a posterior, suboccipital midline approach to resect the posterior arch of C1. This case underscores the importance of differential diagnosis, detailed imaging examinations should be performed to properly assess the stability of the atlantoaxial spine. In patients with neurological symptoms but no severe spinal cord compression, isolated posterior arch should be considered an potential cause of symptoms, and posterior arch resection is effective.

## Introduction

Congenital hypoplasia of the posterior arch of the atlas (C1) is uncommon and typically asymptomatic [1]. Many patients are readily misdiagnosed since the problem is not detected until they undergo imaging following trauma [2, 3]. Cervical high-intensity intramedullary (CHII) lesion is a unique imaging finding, presenting as hyperintense lesions of the spinal cord in the lack of local spinal cord compression [4]. Here, we present a patient with C1 agenesis who had neurological symptoms and a CHII lesion without spinal cord compression or trauma.

## Case report

A 49-year-old woman presented with a 2-year history of recurrent vertigo attacks. One month before admission, she began to have weakness in both lower extremities and unsteady walking, with chest tightness and shortness of breath after walking. She denied any neck pain or weakness, but did mention occasional mild numbness in her hands, particularly on the left side. She did not have any history of trauma or long-term use of any medication. The general and cranial neurological exams were unremarkable. She exhibited no restriction in neck movement, and no clinical worsening was observed when the neck was flexed or extended. She had brisk reflexes in her lower limbs and a positive Hoffmann sign, with no motor or sensory impairments.

**Figure 1:**
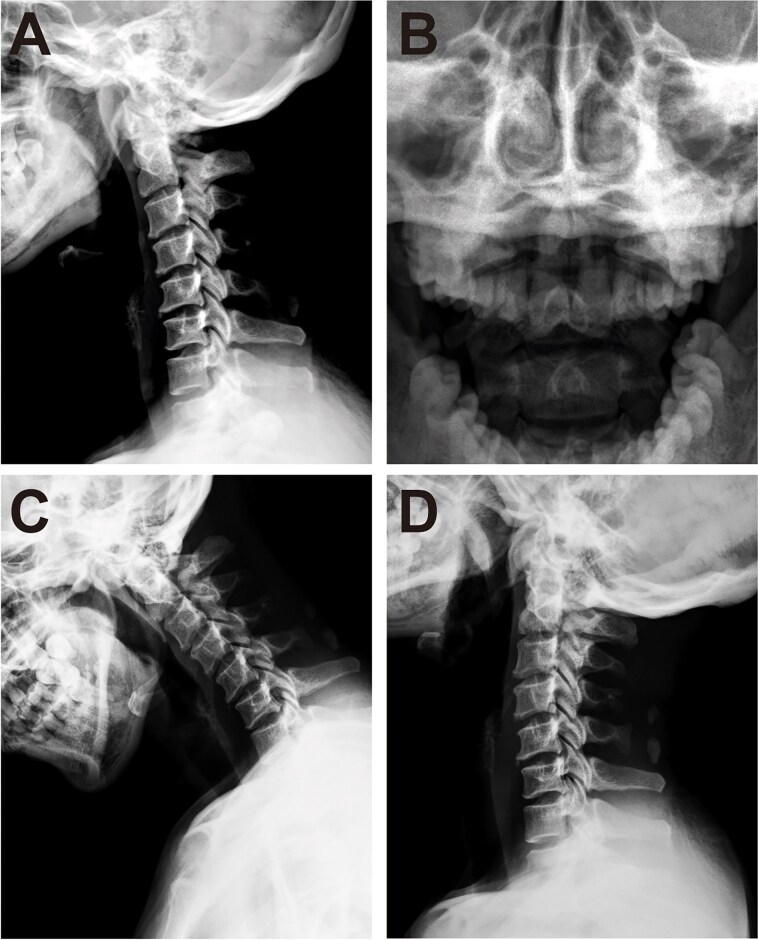
Cervical spine radiograph. (A) Lateral radiograph demonstrating partial aplasia of the posterior arch of the atlas. (B) Open-mouth radiograph showing no atlantoaxial dislocation. (C, D) Cervical spine dynamic X-ray.

**Figure 2:**
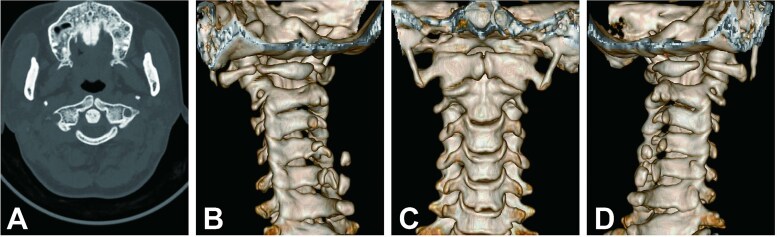
Cervical CT scan. (A) Axial CT image showing hypoplasia of the posterior arch and midline cleft of the anterior arch. (B–D) Three-dimensional reconstructed CT images showing atlas deformity from different views.

**Figure 3:**
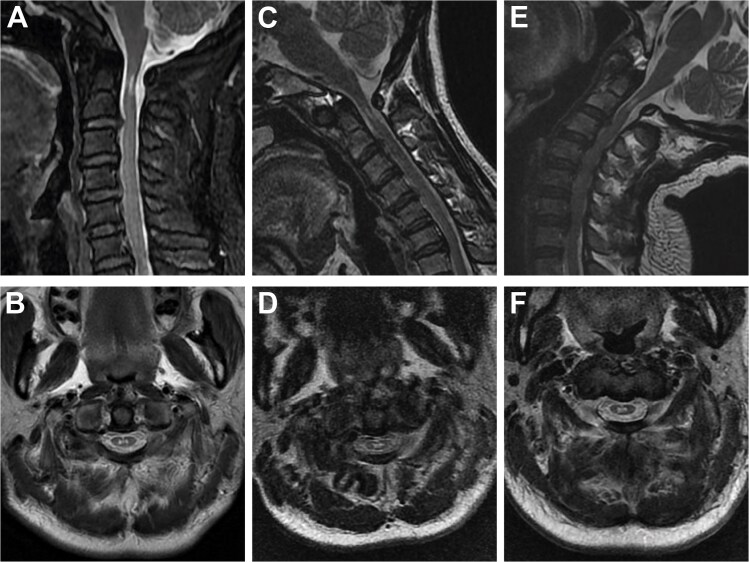
Sagittal and axial T2-weighted MRI of the cervical spine. (A, B) Supine neutral position. (C, D) Supine neck flexion position. (E, F) Supine neck extension position.

Cervical radiographs revealed hypoplasia of C1 but no evidence of atlantoaxial dislocation (Fig. 1). CT scans indicated bilateral bone abnormalities on the lateral sides of the posterior arch, particularly on the left side, with a midline cleft of the anterior arch (Fig. 2A). Three-dimensional reconstruction further demonstrated this abnormality (Fig. 2B–D). T2-weighted MRI revealed an intramedullary high signal at the C1 level, but no compression of the cord was observed (Fig. 3A and B). Cervical flexion-extension imaging indicated minor stenosis of the upper cervical spinal cord in the neck flexion position, but this alteration was not substantial (Fig. 3C–F). We considered that the high signal may be related to the repeated subtle activity of the posterior atlantoaxial arch in the past over a long period of time.

**Figure 4:**
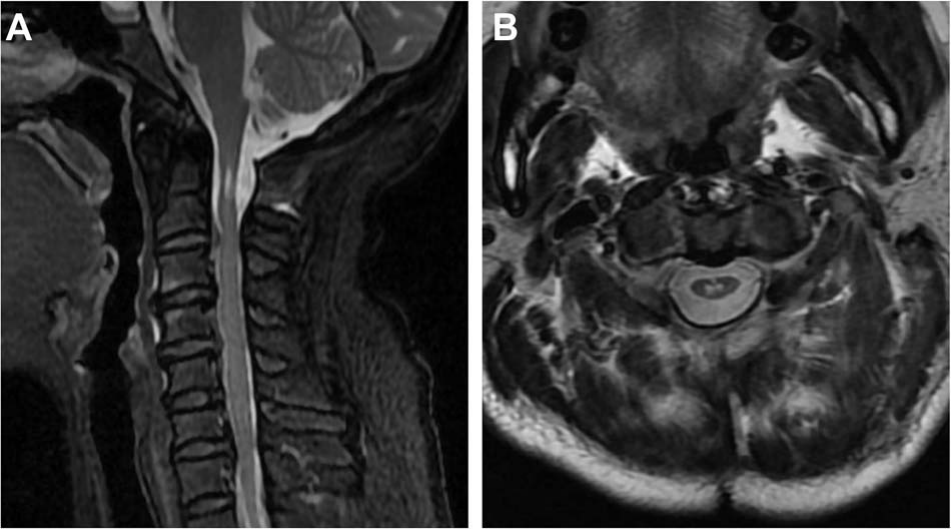
Two-year postoperative MRI results. (A) Sagittal T2-weighted MRI. (B) Axial T2-weighted MRI.

We used a posterior cervical approach to resect the posterior arch of C1. There were no intraoperative complications during the procedure. The patient’s symptoms improved dramatically following the operation. The cervical spinal cord lesion showed no evidence of progression on the 18-month follow-up MRI (Fig. 4A and B). To present, the patient is stable, with no symptoms or signs of recurrence.

Informed consent was obtained from the Institutional Review Board; the patient and his close relatives provided informed consent.

## Discussion

The posterior arch hypoplasia of C1 is not common [1, 5]. Some cases have indicated that this anomaly is not always asymptomatic [6, 7], and the clinical signs include neck pain, symptoms of spinal cord cervical spondylosis, and neurologic deficits following minor trauma [2, 8, 9]. Currarino *et al.* classified this anomaly into five types [10]. Our patient had a type C abnormality with neurological symptoms, CHII lesions, and no spinal cord compression or trauma, which is extremely rare.

The exact cause of C1 arch anomalies is uncertain, but they may be related to a variety of disorders, including cerebral palsy, Turner syndrome, and Klippel–Feil syndrome [11–13]. The atlas has three principal ossification centers: two lateral centers, which form the lateral masses and posterior arch, and an anterior ossification center, which forms the anterior arch [14, 15]. A fourth ossification center is present in rare individuals and subsequently merges with lateral masses to form the posterior arch [16]. A few publications have described multiple main ossification centers in the anterior arch of the atlas [17, 18]. Multiple ossification centers are often symmetrical in size, yet they are occasionally asymmetric [19]. In our patient, a fourth ossification center may have existed, and the anterior arch could have two ossification centers with incomplete fusion.

CHII lesion is a unique imaging finding defined by Van Dijk *et al.* in achondroplasia, presenting as hyperintense lesions of the spinal cord on T2-weighted MRI in the absence of local spinal cord compression [4]. According to Khoo *et al.’s* study, a CHII lesion was found in 47.5% of symptomatic achondroplastic individuals; it appears to be a stable anomaly with no progression or change in morphology [20]. Our case provides evidence for this notion, as the patient’s hyperintense lesions did not change during the 18 months of follow-up after surgery. The finding of CHII lesions in our patient suggests a link between congenital hypoplasia of the atlas and achondroplasia.

Defects of the posterior arch of the atlas are uncommon and often asymptomatic. Its occurrence may be related to the ossification pattern of the atlas and is associated with several diseases. Detailed imaging examinations should be performed to properly assess the stability of the atlantoaxial spine, as well as the spinal cord and associated neural tissues. In patients with neurological symptoms but no severe spinal cord compression, isolated posterior arch should be considered an potential cause of symptoms, and posterior arch resection is effective.
